# Expression Profiling of Exosomal miRNAs Derived from the Peripheral Blood of Kidney Recipients with DGF Using High-Throughput Sequencing

**DOI:** 10.1155/2019/1759697

**Published:** 2019-06-12

**Authors:** Junpeng Wang, Xin Li, Xiaoqiang Wu, Zhiwei Wang, Chan Zhang, Guanghui Cao, Tianzhong Yan

**Affiliations:** ^1^Department of Urology, Henan Provincial People's Hospital, People's Hospital of Zhengzhou University, Zhengzhou 450003, China; ^2^Department of Pathophysiology, School of Basic Medical Sciences, Zhengzhou University, Zhengzhou 450001, China

## Abstract

Delayed graft function (DGF) is one of the major obstacles for graft survival for kidney recipients. It is profound to reduce the incidence of DGF for maintaining long-term graft survival. However, the molecular regulation of DGF is still not adequately explained and the biomarkers for DGF are limited. Exosomes are cell-derived membrane vesicles, contents of which are stable and could be delivered into recipient cells to exert their biological functions. Consequently, exosome-derived proteomic and RNA signature profiles are often used to account for the molecular regulation of diseases or reflect the conditional state of their tissue as biomarkers. Few researches have been done to demonstrate the function of exosomes associated with DGF. In this study, high-throughput sequencing was used to explore the miRNA expression profiling of exosomes in the peripheral blood of kidney recipients with DGF. We identified 52 known and 5 conserved exosomal miRNAs specifically expressed in recipients with DGF. Three coexpressed miRNAs, hsa-miR-33a-5p_R-1, hsa-miR-98-5p, and hsa-miR-151a-5p, were observed to be significantly upregulated in kidney recipients with DGF. Moreover, hsa-miR-151a-5p was positively correlated with the first-week serum CR, BUN, and UA levels of the kidney recipients after transplantation. Furthermore, we also analyzed functions and signaling pathways of the three upregulated miRNAs target genes to uncover putative mechanism of how these exosomal miRNAs functioned in DGF. Overall, these findings identified biomarker candidates for DGF and provided new insights into the important role of the exosomal miRNAs regulation in DGF.

## 1. Introduction

Delayed graft function (DGF) defined as the dialysis requirement in the first week after transplantation is a manifestation of acute renal failure [1]. DGF occurs in as many as 2%-50% of the immediate post-kidney-transplant cases and is a major obstacle for graft survival [2]. A meta-analysis of 34 studies from 1988 through 2007 demonstrated a 49% incidence of acute rejection for patients with DGF compared to 35% incidence for non-DGF patients [1]. In addition, DGF was associated with a 41% and 53% increase in allograft dysfunction and death for patients with DGF, respectively [1, 3]. Thus, it is profound to reduce the incidence of DGF for maintaining long-term graft survival. DGF is commonly considered as a consequence of kidney tubular damage due to ischemia and reperfusion injury (IRI). Moreover, recent studies suggest that the generation of cytotoxic mediators and activation of innate and adaptive immune response could be also correlated to DGF [4]. However, the molecular regulation of DGF is still not adequately explained and the biomarkers for DGF are limited.

Exosomes are cell-derived membrane vesicles (40-100 nm of diameter) present in fluids such as blood, urine, amniotic fluid, breast milk, platelets, synovial fluid, bronchoalveolar lavage fluid, and malignant ascites [5–7]. Exosomes are reported to play a key role in many processes such as cellular activities regulation, intercellular communication, and waste management [6, 8]. Various cell products including protein, DNA, mRNA, and miRNA could be carried by exosomes [9–11]. The contents of exosomes are stable and could be delivered into recipient cells to exert their biological functions; consequently, exosome-derived proteomic and RNA signature profiles are often used to account for the molecular regulation of diseases or reflect the conditional state of their tissue as biomarkers [12–14]. Evidence is accumulating that exosomal contents are also involved in the rejection of transplantation. CD4^+^ CD25^+^ regulatory T cells-derived exosomes could prolong kidney allograft survival in a rat model [15]. Upregulation of exosomal miR-142-3p was also observed during cardiac allograft rejection and it could augment vascular permeability through downregulating the expression of endothelial RAB11FIP2 [16]. In addition, two exosomal proteins (TSPAN1 and HPX) were observed to be significantly higher in patients with acute T cell mediated rejection than in patients without rejection, while exosomal mRNAs transcripts (gp130, CCL4, TNF*α*, CAV1, DARC, and SH2D1B) were helpful to distinguish antibody-mediated rejection patients from cellular rejection recipients [17, 18]. However, few researches have been done to demonstrate the function of exosomes associated with DGF occurring in kidney transplantation.

Therefore, high-throughput sequencing was used to explore the miRNA expression profiling of exosomes in the peripheral blood of kidney recipients with or without DGF and elucidate the regulation of miRNAs in the process of DGF. In this study, we identified a series of known, conserved, and novel exosomal miRNAs in the case of DGF and found that hsa-miR-33a-5p_R-1, hsa-miR-98-5p, and hsa-miR-151a-5p were significantly upregulated in kidney recipients with DGF. Moreover, hsa-miR-33a-5p_R-1 and hsa-miR-151a-5p were positively correlated with the first-week serum CR and BUN levels of the kidney recipients after transplantation, indicating that these two exosomal miRNAs had the potential to be used as biomarkers of DFG in kidney recipients. Furthermore, we also analyzed functions and signaling pathways of the three upregulated miRNAs target genes to uncover putative mechanism of how these exosomal miRNAs function in DGF occurring in kidney transplantation.

## 2. Materials and Methods

### 2.1. Patients and Samples

A total of 9 patients who received donation after cardiac death (DCD) kidney grafts in Provincial People's Hospital of Henan Province were involved in this study. The recipients were divided into two groups: the DGF group and the control group based on whether they needed more than one dialysis within the first week after transplantation or not [19, 20]. Their first-week clinical data after kidney transplantation were collected from Provincial People's Hospital of Henan Province. Clinical data from 5 patients without kidney transplantation were also analyzed as normal controls.

### 2.2. Exosome Isolation

One week after kidney transplantation, exosomes were isolated and purified from the peripheral blood of recipients using the exoEasy Maxi Kit (QIAGEN). Firstly, the blood samples were centrifuged for 10 min at 3000 rpm and 4°C to remove cells and large debris. The upper plasma phase was carefully collected and filtered with a 0.22 *μ*m filter to remove additional cellular fragments. Then larger protein complexes abundant in blood could be removed during the binding and washing steps as follows: (1) mix the filtered sample with XBP buffer (1:1) at room temperature (RT), add the mix onto the exoEasy spin column, centrifuge at 500 × g for 1 min, and discard the flow-through and place the column back into the same collection tube; (2) add 10 mL XWP buffer, centrifuge at 5000 × g for 5 min, and discard the flow-through together with the collection tube. Finally, transfer the spin column to a fresh collection tube, add 400 *μ*l XE buffer to the membrane and incubate for 1 min, and centrifuge at 5000 × g for 5 min to collect exosomes in the eluate.

### 2.3. Nanoparticle Tracking Analysis and Transmission Electron Microscopy

The concentration and size distribution of the isolated exosomes were detected by ZetaView (Particle Metrix, Germany) and analyzed using the in-build nanoparticle tracking analysis (NTA) software ZetaView 8.02.31. A 20 *μ*L aliquot that contained about 4-5 × 10^6^ particles was fixed with 1% glutaraldehyde and loaded onto a carbon-coated grid for 2 min at room temperature. The grid was negatively stained with 2% aqueous phosphotungstic acid for 1 min and then imaged under a transmission electron microscope (JEM-1400, Japan).

### 2.4. Western Blot

Exosomal lysates were prepared using RIPA buffer with protease inhibitor mixture (Bi Yuntian, China), and protein concentration was determined using Pierce BCA Protein Assay Kit (Thermo Scientific, USA). About 20 *μ*g of exosomal lysates from each sample in both DGF and control groups was loaded onto SDS-polyacrylamide gels and then transferred onto polyvinylidene difluoride membranes (0.45 *μ*m pore size, Millipore, USA). The blots were blocked in 5% non-fat dry milk for 1 h at room temperature. After blocking, the membranes were incubated overnight at 4°C with primary rabbit anti-CD63 antibody (1:1000; ab68418, Abcam, USA), rabbit anti-CD9 antibody (1:1000; ab92726, Abcam, USA), or rabbit anti-*β*-actin antibody (1:1000; ab8227, Abcam, USA). After being washed by PBST, the membranes were incubated with HRP conjugated anti-rabbit IgG (1:20,000; Thermo Scientific, USA) for 1 h at 37°C. Subsequently, blots were developed using Lumi-Light Western Blotting Substrate (Roche, Switzerland), imaged, and quantified using ChemiDoc MP imaging system (Bio-Rad, USA).

### 2.5. RNA Extraction

Total RNAs from exosomes were isolated using the mirVana miRNA Isolation Kit (Ambion, USA) according to the manufacturer's protocol. The RNA quality and concentration were assessed with the OD ratio 260/280 using a Nanodrop 1000 (Thermo Scientific, USA).

### 2.6. miRNA Libraries Construction and Sequencing

TruSeq MiRNA Sample Prep Kit (Illumina, USA) was used to construct miRNA sequencing libraries according to the manufacturer's protocol. The 3′- and 5′-adapter were ligated to approximately 1 *μ*g of total RNA from each sample using T4 RNA ligase (Epicentre, USA) at 28°C for 1 h, respectively. The adapter-ligated miRNAs were dissolved in RNase free water and used as templates for RT-PCR to create and amplify the cDNA constructs. Then the PCR-amplified cDNA products from the 6% TBE PAGE gels were extracted and then purified. After the completed libraries were evaluated with a quantitative real-time PCR to ensure acceptable quality and confirm that adapters were correctly added, the high-throughput sequencing of the cDNA was done on the HiSeq 2500 (Illumina, USA). Image analysis and base calling were performed with the ACGT101-miR software (LC Sciences, USA).

### 2.7. miRNA Target Prediction, Functional Annotation, and Pathway Enrichment Analysis

Two algorithms, miRanda and TargetScan, were used to predict the target genes of exosomal miRNAs which were differentially expressed between DGF and control groups [21, 22]. Only the miRNA targets identified by both miRanda and TargetScan algorithms composed the final predicted targets. Subsequently, the gene ontology terms (http://www.geneontology.org/) and KEGG pathway terms (http://www.genome.jp/kegg) enriched in predicted target genes were determined to explore the function and related pathway of the targets [23].

### 2.8. Ethics Statement

Written informed consent was obtained from all patients. This study was approved by the Protection of Human Subjects Committee, Provincial People's Hospital of Henan Province.

### 2.9. Statistical Analysis of Data

Statistical assessment was performed using SPSS 19.0 for Windows. Firstly, we conducted the Shapiro-Wilk normality test to assess whether continuous data have been drawn from a normally distributed population. Since all continuous data were normally distributed, they were assessed by one-way ANOVA or* t*-test. Categorical data were evaluated by nonparametric test. Pearson correlation was used for correlation analysis miRNA expressions and the first-week serum CR or BUN levels from the kidney recipients.* P* < 0.05 was considered as statistically significant.

## 3. Results

### 3.1. Basic Information and Clinical Data of Kidney Recipients with DGF

Nine patients who received donation after cardiac death (DCD) kidney grafts were involved in this study. Immunosuppressive treatment with mycophenolate mofetil, prednisone and tacrolimus, and induction therapy with thymoglobuline were given to the recipients after transplantation for preventing acute rejection. The basic information of these kidney recipients was shown in Table 1. One week after transplantation, the blood and urine samples from the recipients were collected and then their serum creatinine (CR), blood urea nitrogen (BUN), uric acid (UA), hemoglobin (HGB), albumin (ALB), platelet (PLT), and urine protein (Pro) levels were detected. We divided the recipients into two groups: those with delayed graft function (DGF, n = 4) and those with immediate graft function, considered as the control group (n = 5), and found that the CR levels, BUN levels, and UA levels of the recipients in DGF group were significantly higher than those of the recipients in control group, respectively (CR:* P* = 0.0002; BUN:* P* < 0.0001; UA:* P* = 0.0120; Table 2). Clinical data from patients without kidney transplantation (n = 5; basic information shown in Table 1) were also analyzed as normal controls. When compared with patients without kidney transplantation, recipients in DGF group exhibited significantly higher levels of CR and BUN (CR:* P* = 0.0002; BUN:* P* < 0.0001; Table 2), whereas clinical data of kidney recipients in control group and patients without kidney transplantation were similar (Table 2).

### 3.2. Characterization of Exosomes in Peripheral Blood of Recipients after Kidney Transplantation

**Figure 1:**
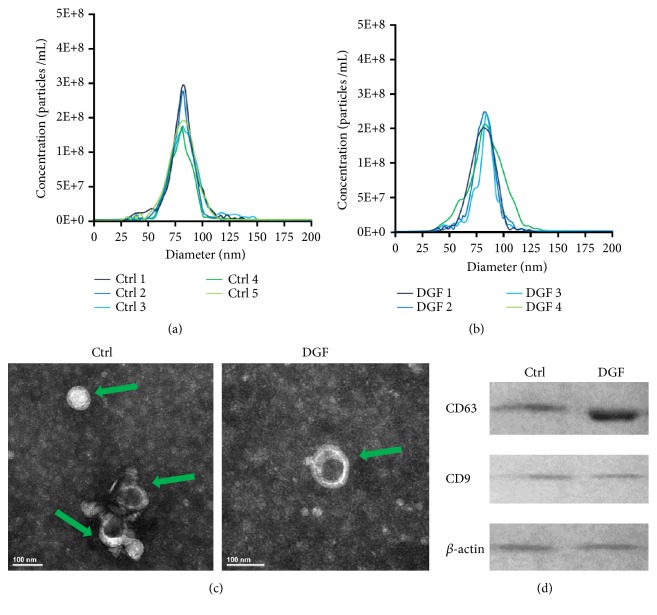
*Identification of exosomes through nanoparticle tracking analysis, transmission electron microscopy, and Western blot*. (a) Concentration and size of exosomes in control (a) and DGF (b) groups were analyzed by the nanoparticle tracking analysis. (c) Exosomes isolated from the peripheral blood of recipients one week after kidney transplantation have a saucer-like shape that was limited by a lipid bilayer. Green arrowheads point to exosomes. Scale bar = 100 nm. (d) Exosomes are positive for CD63 and CD9, as determined through Western blot analysis. *β*-Actin was used as a positive control.

Based on the unique size and density, exosomes were isolated from the peripheral blood of recipients in both DGF and control groups one week after kidney transplantation using the exoEasy Maxi Kit (QIAGEN). Then the exosomes were identified through nanoparticle tracking analysis, transmission electron microscopy, and Western blot. The results showed that small vesicles with diameters ranging from approximately 60 to 90 nm and a lipid bilayer could be observed under the transmission electron microscopy (Figures 1(a), 1(b), and 1(c)). Moreover, Western blot analysis confirmed presence of the known exosomal membrane proteins, CD63 and CD9 (Figure 1(d)). These results demonstrated that the small vesicles we isolated were exosomes.

### 3.3. Identification of Exosomal miRNAs

**Figure 2:**
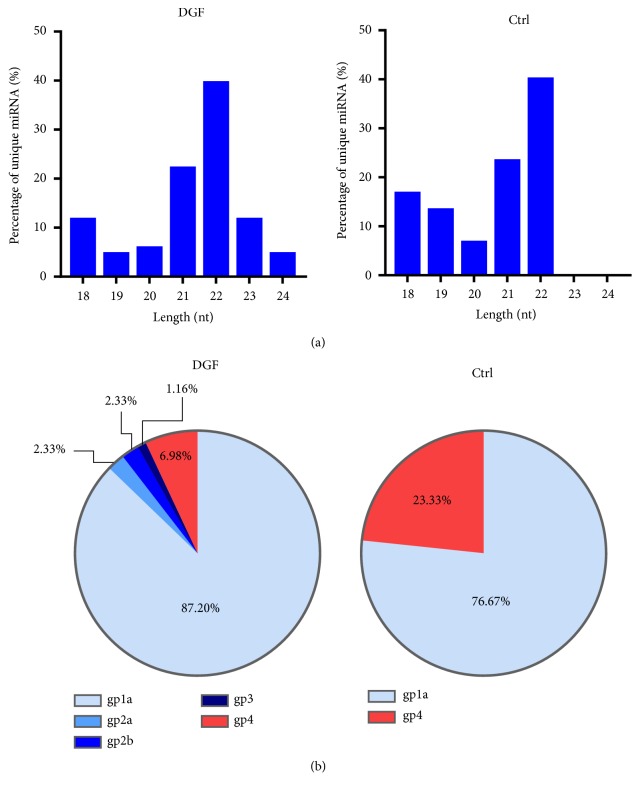
*Length and category distribution of sequenced exosomal miRNAs in the peripheral blood of kidney recipients*. (a) Category distribution of sequenced exosomal miRNAs. Reads with length 18–26 nt were chosen for mapping. Length distribution of sequenced miRNAs is shown. The nucleotide (nt) lengths of cloned miRNAs are shown on x-axis; the number of total reads by deep sequencing is shown on y-axis. (b) Category distribution of sequenced exosomal miRNAs. Gp1a: reads were mapped to miRNAs/pre-miRNAs of specific species in miRBase and the pre-miRNAs were further mapped to genome and EST. gp2a: reads were mapped to miRNAs/pre-miRNAs of selected species in miRBase and the mapped pre-miRNAs were not further mapped to genome, but the reads (and of course the miRNAs of the pre-miRNAs) were mapped to genome. The extended genome sequences from the genome loci may form hairpins. gp2b: reads were mapped to miRNAs/pre-miRNAs of selected species in miRBase and the mapped pre-miRNAs were not further mapped to genome, but the reads (and of course the miRNAs of the pre-miRNAs) were mapped to genome. The extended genome sequences from the genome loci may not form hairpins. gp3: reads were mapped to miRNAs/pre-miRNAs of selected species in miRBase and the mapped pre-miRNAs were not further mapped to genome, and the reads were not mapped to genome either. But the reads were mapped to the miRNAs (Matures). gp4: reads were not mapped to pre-miRNAs of selected species in miRBase. But the reads were mapped to genome and the extended genome sequences from genome may form hairpins.

A cDNA library of miRNAs was constructed from the exosomes in the peripheral blood of kidney recipients one week after the transplantation. After removing low-quality reads, contaminants, and adaptors, we obtained 87 unique miRNAs of 18 and 26 bases in length through Solexa sequencing. To guarantee the accuracy of sequence data, only the sequences that were observed in all samples from DGF or control group were selected as miRNA molecules (Table 3). The length distribution of unique exosomal miRNAs (18–24 nt) varied in the DGF and control groups. The lengths of miRNAs in the DGF group ranged from 18 to 24 nt (Figure 2(a), left panel), while the length of the miRNAs in the control group ranged from 18 to 22 nt (Figure 2(b), right panel). The most abundant size class in both groups was 22 nt, followed by 21 nt and then by 18 nt (Figure 2). To further identify the unique miRNA we obtained by high-throughput sequencing, all the unique miRNA clean reads were compared with the known human miRNAs in miRBase 18.0 (University of Manchester, Manchester, UK). Among the 87 unique miRNAs we identified, 86 and 30 miRNAs could be detected in the DGF and control groups, respectively. The known (gp1a), conserved (gp2a, gp2b, and gp3), and novel (gp4) miRNAs accounted for, respectively, 87.2%, 5.82%, and 6.98% of the total miRNAs in the DGF group (Figure 2(b), left panel), while known and novel miRNAs separately made up 76.67% and 23.33% of the total miRNAs in the control group (Figure 2(b), right panel). In conclusion, these results demonstrated that the exosomal miRNA profiling in the DGF and control group is significantly different.

### 3.4. Analysis of Differentially Expressed Exosomal miRNAs in DGF and Control Groups

**Figure 3:**
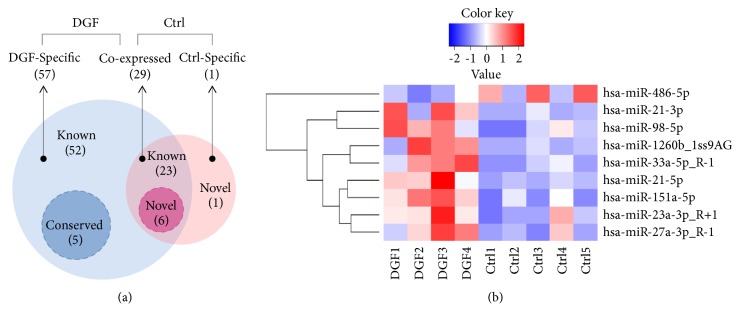
*Analysis of differentially expressed exosomal miRNAs in DGF and control groups*. (a) Venn diagram of differentially expressed exosomal miRNAs in DGF (blue) and control (red) groups. (b) Heat map of the differentially expressed exosomal miRNAs in DGF and control groups. The color of each pattern represents the fold change as log⁡2, from high (red) to low (blue).

We summarized the coexpressed and specific exosomal miRNA sequences between the DGF and control group. Among the exosomal miRNAs we identified, 29 miRNAs were shared in both the DGF and control groups, while 57 miRNAs containing 52 known miRNAs and 5 conserved miRNAs and 1 novel miRNA were specifically expressed in the DGF and control groups, respectively (Figure 3(a)). Moreover, we analyzed the expressions of the 29 coexpressed miRNAs in the DGF and control group (Figure 3(a)). The coexpressed miRNAs were composed of 23 known miRNAs and 6 novel miRNAs (Figure 3(a)). Among the known coexpressed exosomal miRNAs, 3 miRNAs, hsa-miR-33a-5p_R-1, hsa-miR-98-5p, and hsa-miR-151a-5p, were detected at noticeably higher levels in the DGF group than that in the control group (*P* = 0.007, 0.024 and 0.027, resp.; fold change > 2; Figure 3(b)). The detailed information of the differentially expressed exosomal miRNAs was expressed in Table 4. These results suggested that the exosomal miRNAs, hsa-miR-33a-5p_R-1, hsa-miR-98-5p, and hsa-miR-151a-5p, had the potential to be the biomarkers for DGF.

### 3.5. Correlation Analysis of Differentially Expressed Exosomal miRNAs with Clinical Parameters

**Figure 4:**
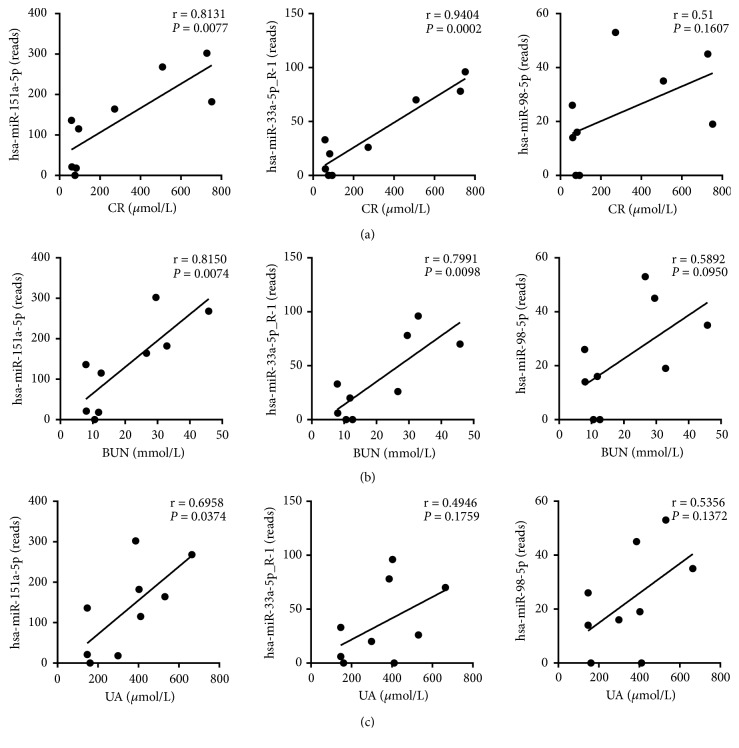
*Correlation analysis of differentially expressed exosomal miRNAs with clinical parameters*. Pearson correlation was performed to examine the relationship between hsa-miR-151a-5p, hsa-miR-33a-5p_R-1, and hsa-miR-98-5p and the first-week serum (a) CR, (b) BUN, or (c) UA levels in the kidney recipients after transplantation. Pearson correlation coefficient (r) and *P* value are plotted in each case.

Since the CR, BUN, or UA levels of the kidney recipients in the DGF and control group were significantly different, the correlation analysis of the hsa-miR-33a-5p_R-1, hsa-miR-98-5p, or hsa-miR-151a-5p miRNA expressions with these clinical parameters was conducted to further assess the potential of the three exosomal miRNAs to be the diagnostic indicators of DGF. Noticeably, the expression of hsa-miR-151a-5p was positively correlated with CR (r = 0.8131,* P* = 0.0077; Figure 4(a)), BUN (r = 0.8150,* P* = 0.0074; Figure 4(b)), and UA (r = 0.6958,* P* = 0.0374; Figure 4(c)) levels, while the expressions of hsa-miR-33a-5p_R-1 only showed significant positive correlation with the CR (r = 0.9404,* P* = 0.0002; Figure 4(a)) and BUN (r = 0.7991,* P* = 0.0098; Figure 4(b)) levels. No significant correlation existed between the hsa-miR-98-5p expression and the CR, BUN, and UA levels (Figures 4(a), 4(b), and 4(c)). These results demonstrated that hsa-miR-151a-5p was not only a biomarker candidate for DGF but could reflect the CR, BUN, and UA levels.

### 3.6. Functional Analysis of Differentially Expressed miRNA Target Genes

**Figure 5:**
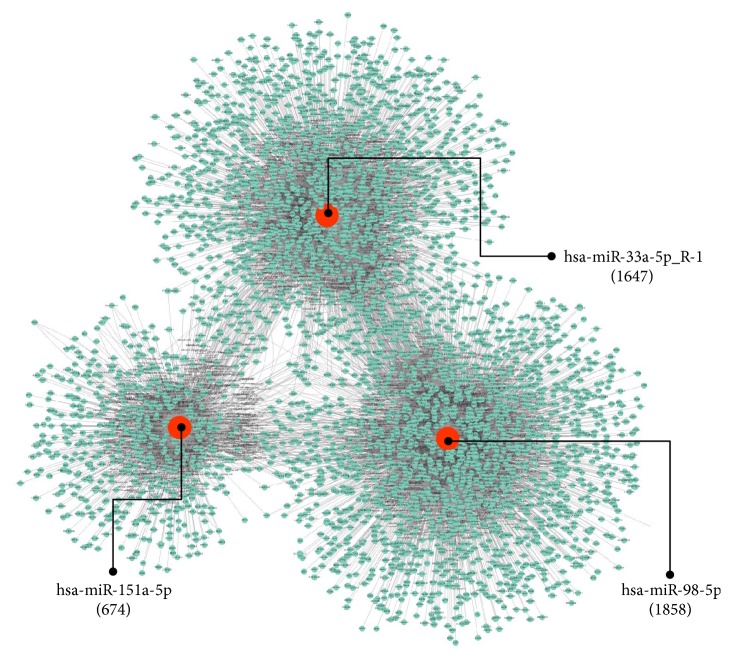
*The regulatory network between miRNAs and target genes associated with DGF*. The red and green roundness represented the miRNAs and genes, respectively. The target gene number was expressed in parentheses.

**Figure 6:**
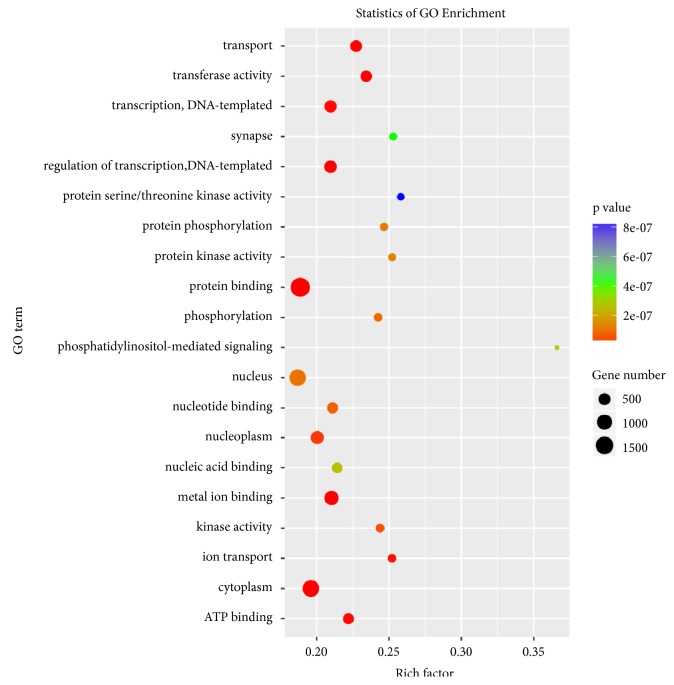
*Go analysis of target genes of the predicted differentially expressed miRNAs*. Advanced bubble chart shows enrichment of differentially expressed genes in GO terms. y-axis represented pathway, and x-axis represented rich factor, which meant the ratio of the amount of differentially expressed genes enriched in the pathway and the amount of all genes annotated in this pathway. Size and color of the bubble represented the amount of differentially expressed genes enriched in the pathway and enrichment significance, respectively.

**Figure 7:**
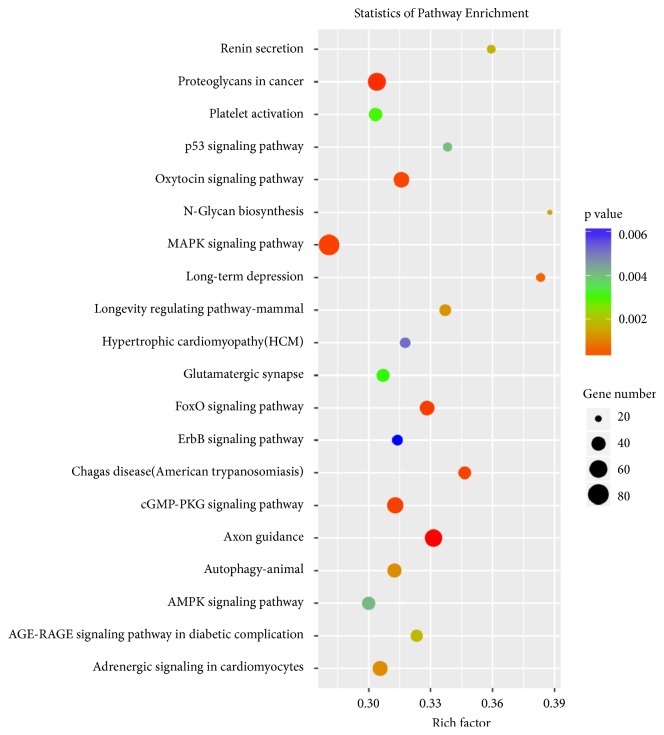
*KEGG pathways significantly enriched in the predicted target genes of differentially expressed miRNAs*. Advanced bubble chart shows enrichment of differentially expressed genes in signaling pathways. y-axis represented pathway, and x-axis represented rich factor, which meant the ratio of the amount of differentially expressed genes enriched in the pathway and the amount of all genes annotated in this pathway. Size and color of the bubble represented the amount of differentially expressed genes enriched in the pathway and enrichment significance, respectively.

The target genes of the three differentially expressed exosomal miRNAs, hsa-miR-33a-5p_R-1, hsa-miR-98-5p, or hsa-miR-151a-5p, were predicted using TargetScan and miRanda software. 1647, 1858, and 647 target genes of hsa-miR-33a-5p_R-1, hsa-miR-98-5p, and hsa-miR-151a-5p were identified, respectively (Figure 5). To further highlight the functional features of exosomal miRNAs, the putative target genes were subsequently annotated in GO terms. As shown in Figure 6, the target genes were mainly enriched in “protein binding” (1850 genes,* P *= 9.807E-15), “cytoplasm” (1,307 genes,* P* = 3.997E-14), “nucleus” (1,275 genes,* P* = 1.027E-07), “cytosol” (971 genes,* P* = 1.158E-05), “metal ion binding” (825 genes,* P* = 4.330E-15), “nucleoplasm” (665 genes,* P* = 2.626E-08), “regulation of transcription, DNA-templated” (573 genes,* P* = 3.456E-10), “transcription, DNA-templated” (532 genes,* P* = 1.681E-09), and so forth (Figure 6). KEGG enrichment analysis demonstrated that target genes were significantly enriched in 52 signaling pathways. Among these signaling pathways, “MAPK signaling pathway” (82 genes,* P* = 1.158E-05), “Proteoglycans in cancer” (62 genes,* P* = 1.158E-05), “Axon guidance” (58 genes,* P* = 1.158E-05), “cGMP - PKG signaling pathway” (51 genes,* P* = 1.158E-05), and “Oxytocin signaling pathway” (48 genes,* P* = 1.158E-05) were the five most significantly enriched signaling pathways (Figure 7).

## 4. Discussion

Since few researches have been done to demonstrate the role of exosomal miRNAs associated with the process of DGF in kidney transplantation, in this study, we utilized high-throughput sequencing to explore the miRNA expression profiling and function of exosomes in the peripheral blood of kidney recipients with or without DGF. The lengths of the miRNAs we identified in the DGF group ranged from 18 to 24 nt, while the length of the miRNAs in the control group ranged from 18 to 22 nt. Though the length distributions of unique miRNAs varied in the DGF and control groups, they exhibited the canonical size range distribution common to mammalian miRNAs [24]. Through comparing the miRNA sequences with the known miRNAs in the miRBase, we identified 87 exosomal miRNAs in the peripheral blood of kidney recipients with DGF after transplantation.

Among the 87 exosomal miRNAs, 57 were specific and were expressed specially in the recipients with DGF, while 29 exosomal miRNAs were coexpressed in the DGF and control groups. Three coexpressed exosomal miRNAs, hsa-miR-33a-5p_R-1, hsa-miR-98-5p, and hsa-miR-151a-5p, were further found to be significantly upregulated in the peripheral blood of kidney recipients with DGF, indicating that these exosomal miRNAs could be biomarker candidates for DGF. Mean values of CR, BUN, and UA were observed to be significantly increased in the liver recipients with (acute kidney injury) AKI at postoperative days 1, 3, and 7 [25]. In our study, the CR, BUN, and UA levels of the recipients in DGF group were also significantly higher than those of the recipients in control group, respectively. It was remarkable that the hsa-miR-151a-5p expression showed significant positive correlation with CR, BUN, and UA levels of the patients as well. Thus, compared with two other exosomal miRNAs, hsa-miR-151a-5p seemed to have more clinical value, for it could not only be a biomarker candidate for DGF but also reflect CR, BUN, and UA more accurately.

Few evidences in previous research were provided to demonstrate the relationship between kidney transplantation and hsa-miR-33a-5p_R-1, hsa-miR-98-5p, or hsa-miR-151a-5p. In this study, GO and KEGG pathway enrichment analyses were utilized to reveal biological processes and functions of differentially expressed exosomal miRNA target genes relevant for DGF. Our results indicated that the differentially expressed genes were mainly involved in MAPK signaling pathway and biological processes including DNA-templated transcription. MAPK signaling pathway was shown to play a pivotal role in chronic allograft dysfunction of kidney transplant recipients by regulating kidney interstitial fibrosis. Renal fibrosis was ameliorated by interfering the MAPK signaling pathway in an animal model of unilateral ureteral obstruction [26–28]. P38 MAPK signaling pathway could be observed to be activated in human kidney (HK-2) cells stimulated by IL-33 and kidney recipients with chronic allograft dysfunction [29]. Recent studies also uncovered that MAPK signaling pathway was associated with cerebral, cardiac, and retinal ischemia reperfusion injury [30–32]. DGF is primarily a consequence of ischemia and reperfusion (IR) injury resulting in postischemic acute tubular necrosis [2]. Therefore, probably, the differentially expressed exosomal miRNAs, hsa-miR-33a-5p_R-1, hsa-miR-98-5p, and hsa-miR-151a-5p, might moderate the process of DGF by regulating the MAPK signaling pathway associated with ischemia reperfusion injury.

Noticeably, two DGF-specific exosomal miRNAs that we identified, hsa-miR-20a-5p and hsa-miR-17-5p, were reported also to be significantly upregulated in the biopsy specimens of DGF kidneys with acute tubular necrosis [33]. DGF is regarded as a consequence of ischemia and reperfusion injury (IR) resulting in postischemic acute tubular necrosis [2]. Essentially, these results were consistent with ours and further confirmed the possibility of exosomal hsa-miR-20a-5p and hsa-miR-17-5p in the peripheral blood of the kidney recipients to be the biomarkers of DGF. hsa-miR-17-5p was also found to be involved in liver allograft rejection by regulating PD-L1 expression [34], suggesting that the regulation of PD-L1 may be a possible mechanism for hsa-miR-17-5p to function in DGF. However, the studies about the role of hsa-miR-20a-5p in kidney transplantation were very limited; most researches about hsa-miR-20a-5p are associated with cancer [35, 36]. Though significant upregulation of exosomal miRNAs hsa-miR-21-5p and hsa-miR-106b-5p could be detected in DGF kidney in the previous study [33], our results demonstrated that the expressions of these two exosomal miRNAs were similar in the peripheral blood of the kidney recipients with or without DGF.

Overall, in this study, we identified the expression profiling of exosomal miRNAs in the peripheral blood of kidney recipients with DGF using Solexa high-throughput sequencing and provided new insights into the important role of the exosome-derived miRNA regulation in DGF. The miRNA candidates, hsa-miR-33a-5p_R-1, hsa-miR-98-5p, and hsa-miR-151a-5p, which were upregulated in the process of DGF, have the potential to be used as biomarkers to predict graft function in the kidney recipients and the inhibitors of these miRNAs might offer a novel treatment option in transplantation.

## Figures and Tables

**Table 1:** Basic information of the kidney recipients.

Patient ID	Height (cm)	Weight (kg)	Age	Gender	Type of transplantation	Induction therapy	Maintenance immunosuppressive drugs
DGF 1	178	78	52	Male	DCD	Thymoglobuline	Tac+MMF+Pred
DGF 2	170	72	48	Male	DCD	Thymoglobuline	Tac+MMF+Pred
DGF 3	171	62	51	Male	DCD	Thymoglobuline	Tac+MMF+Pred
DGF 4	163	50	24	Male	DCD	Thymoglobuline	Tac+MMF+Pred
Ctrl 1	169	67.5	38	Male	DCD	Thymoglobuline	Tac+MMF+Pred
Ctrl 2	180	71	32	Male	DCD	Thymoglobuline	Tac+MMF+Pred
Ctrl 3	160	42	40	Female	DCD	Thymoglobuline	Tac+MMF+Pred
Ctrl 4	168	55	40	Male	DCD	Thymoglobuline	Tac+MMF+Pred
Ctrl 5	155	48	29	Female	DCD	Thymoglobuline	Tac+MMF+Pred
Ntr 1	173	83	51	Male	-	-	-
Ntr 2	172	72	44	Male	-	-	-
Ntr 3	160	69	37	Female	-	-	-
Ntr 4	166	64	32	Male	-	-	-
Ntr 5	173	70	26	Male	-	-	-

DGF: delayed graft function; Ctrl: control; Ntr: normal control (patients without kidney transplantation); DCD: donation after cardiac death; Tac: tacrolimus; MMF: mycophenolate mofetil; Pred: prednisone.

**Table 2:** First-week clinical data of the kidney recipients after transplantation.

Variable	DGF	Ctrl	Ntr	*P1*	*P2*	*P3*
CR (*μ*mol/L)	565.30 ± 224.00	74.40 ± 14.67	71.60 ± 14.55	0.0002	0.0002	0.9992
BUN (mmol/L)	33.70 ± 8.47	10.18 ± 2.16	6.16 ± 1.14	<0.0001	<0.0001	0.3914
UA (*μ*mol/L)	496.00 ± 129.40	233.00 ± 118.30	332.80 ± 85.66	0.0120	0.1167	0.3640
HGB (g/L)	91.50 ± 13.80	109.80 ± 22.53	130.20 ± 25.34	0.4459	0.0535	0.3336
ALB (g/L)	36.15 ± 1.97	38.96 ± 4.50	43.30 ± 4.46	0.5576	0.0506	0.2364
PLT (×10^9^/L)	157.80 ± 58.59	122.00 ± 53.37	202.60 ± 43.35	0.5718	0.4253	0.0734
Pro, -/+/++/+++	1/0/1/2	3/0/1/1	5/0/0/0	0.7890^*a*^	0.0735^*a*^	0.6924^*a*^

Data were expressed as mean antibody concentration ± SD. *P1, P2*, and *P3* values referred to the comparison of the patients between DGF group and Ctrl group, DGF group and Ntr group, and DGF group and Ntr group, respectively. ^*a*^Nonparametric test. DGF: delayed graft function; Ctrl: control; Ntr: normal control (patients without kidney transplantation); CR: serum creatinine; BUN: blood urea nitrogen; UA: uric acid; HGB: hemoglobin; ALB: albumin; PLT: platelet; Pro: urine protein.

**Table 3:** Exosomal miRNAs identified in the peripheral blood of kidney recipients.

miRNA name	Length	Sequence	Group	Expression level	Common/specific
hsa-let-7a-5p	22	TGAGGTAGTAGGTTGTATAGTT	gp1a	high	DGF specific
hsa-let-7b-5p	22	TGAGGTAGTAGGTTGTGTGGTT	gp1a	high	common
hsa-let-7d-5p	22	AGAGGTAGTAGGTTGCATAGTT	gp1a	high	DGF specific
hsa-let-7f-5p	22	TGAGGTAGTAGATTGTATAGTT	gp1a	high	DGF specific
hsa-let-7g-5p	22	TGAGGTAGTAGTTTGTACAGTT	gp1a	high	DGF specific
hsa-let-7i-5p_R-1	21	TGAGGTAGTAGTTTGTGCTGT	gp1a	high	common
hsa-miR-100-5p_R-1	21	AACCCGTAGATCCGAACTTGT	gp1a	middle	DGF specific
hsa-miR-101-3p_R+1	22	TACAGTACTGTGATAACTGAAG	gp1a	high	common
hsa-miR-103a-3p_R-2	21	AGCAGCATTGTACAGGGCTAT	gp1a	high	common
hsa-miR-106a-5p_1ss1AC	23	CAAAGTGCTTACAGTGCAGGTAG	gp1a	high	DGF specific
hsa-miR-106b-5p_R-1	20	TAAAGTGCTGACAGTGCAGA	gp1a	high	common
hsa-miR-107_R-2	21	AGCAGCATTGTACAGGGCTAT	gp1a	high	common
hsa-miR-10b-5p_R-1	22	TACCCTGTAGAACCGAATTTGT	gp1a	middle	DGF specific
hsa-miR-125b-5p	22	TCCCTGAGACCCTAACTTGTGA	gp1a	high	DGF specific
hsa-miR-126-3p	22	TCGTACCGTGAGTAATAATGCG	gp1a	high	common
hsa-miR-126-5p	21	CATTATTACTTTTGGTACGCG	gp1a	high	DGF specific
hsa-miR-128-3p	21	TCACAGTGAACCGGTCTCTTT	gp1a	middle	DGF specific
hsa-miR-142-3p_L-1	22	GTAGTGTTTCCTACTTTATGGA	gp1a	high	DGF specific
hsa-miR-142-5p_L+2R-3	20	CCCATAAAGTAGAAAGCACT	gp1a	high	DGF specific
hsa-miR-143-3p_R+1	22	TGAGATGAAGCACTGTAGCTCT	gp1a	high	DGF specific
hsa-miR-144-3p_R-1	19	TACAGTATAGATGATGTAC	gp1a	high	common
hsa-miR-145-5p	23	GTCCAGTTTTCCCAGGAATCCCT	gp1a	middle	DGF specific
hsa-miR-146a-5p	22	TGAGAACTGAATTCCATGGGTT	gp1a	middle	DGF specific
hsa-miR-148a-3p	22	TCAGTGCACTACAGAACTTTGT	gp1a	high	DGF specific
hsa-miR-148b-3p	22	TCAGTGCATCACAGAACTTTGT	gp1a	middle	DGF specific
hsa-miR-150-5p	22	TCTCCCAACCCTTGTACCAGTG	gp1a	middle	DGF specific
hsa-miR-151a-5p	21	TCGAGGAGCTCACAGTCTAGT	gp1a	middle	DGF specific
hsa-miR-152-3p	21	TCAGTGCATGACAGAACTTGG	gp1a	middle	DGF specific
hsa-miR-15a-5p_R-1	21	TAGCAGCACATAATGGTTTGT	gp1a	high	common
hsa-miR-15b-5p_R-3	19	TAGCAGCACATCATGGTTT	gp1a	high	common
hsa-miR-16-5p	22	TAGCAGCACGTAAATATTGGCG	gp1a	high	common
hsa-miR-17-5p	23	CAAAGTGCTTACAGTGCAGGTAG	gp1a	high	DGF specific
hsa-miR-181a-5p	23	AACATTCAACGCTGTCGGTGAGT	gp1a	middle	DGF specific
hsa-miR-185-5p	22	TGGAGAGAAAGGCAGTTCCTGA	gp1a	middle	DGF specific
hsa-miR-191-5p	23	CAACGGAATCCCAAAAGCAGCTG	gp1a	high	DGF specific
hsa-miR-199a-3p	22	ACAGTAGTCTGCACATTGGTTA	gp1a	middle	DGF specific
hsa-miR-19b-3p_R-2	21	TGTGCAAATCCATGCAAAACT	gp1a	middle	DGF specific
hsa-miR-20a-5p	23	TAAAGTGCTTATAGTGCAGGTAG	gp1a	high	DGF specific
hsa-miR-21-5p	22	TAGCTTATCAGACTGATGTTGA	gp1a	high	common
hsa-miR-221-3p_R-1	22	AGCTACATTGTCTGCTGGGTTT	gp1a	high	DGF specific
hsa-miR-223-3p_R+1	23	TGTCAGTTTGTCAAATACCCCAA	gp1a	high	DGF specific
hsa-miR-22-3p	22	AAGCTGCCAGTTGAAGAACTGT	gp1a	high	common
hsa-miR-22-5p_R-1	21	AGTTCTTCAGTGGCAAGCTTT	gp1a	middle	DGF specific
hsa-miR-23a-3p_R+1	22	ATCACATTGCCAGGGATTTCCA	gp1a	high	DGF specific
hsa-miR-23b-3p_R-5	18	ATCACATTGCCAGGGATT	gp1a	middle	DGF specific
hsa-miR-24-3p	22	TGGCTCAGTTCAGCAGGAACAG	gp1a	middle	DGF specific
hsa-miR-25-3p	22	CATTGCACTTGTCTCGGTCTGA	gp1a	high	common
hsa-miR-26a-5p	22	TTCAAGTAATCCAGGATAGGCT	gp1a	high	common
hsa-miR-26b-5p_R+1	22	TTCAAGTAATTCAGGATAGGTT	gp1a	high	common
hsa-miR-27a-3p_R-1	20	TTCACAGTGGCTAAGTTCCG	gp1a	high	common
hsa-miR-27b-3p	21	TTCACAGTGGCTAAGTTCTGC	gp1a	high	DGF specific
hsa-miR-29a-3p_R-1_1ss10CT	21	TAGCACCATTTGAAATCGGTT	gp1a	high	common
hsa-miR-29c-3p_R-1	21	TAGCACCATTTGAAATCGGTT	gp1a	high	common
hsa-miR-30a-5p_R+2	24	TGTAAACATCCTCGACTGGAAGCT	gp1a	high	DGF specific
hsa-miR-30b-5p	22	TGTAAACATCCTACACTCAGCT	gp1a	high	DGF specific
hsa-miR-30c-5p_R+1	24	TGTAAACATCCTACACTCTCAGCT	gp1a	high	DGF specific
hsa-miR-30d-5p_R+2	24	TGTAAACATCCCCGACTGGAAGCT	gp1a	high	DGF specific
hsa-miR-30e-5p_R+2	24	TGTAAACATCCTTGACTGGAAGCT	gp1a	high	DGF specific
hsa-miR-320a-3p	22	AAAAGCTGGGTTGAGAGGGCGA	gp1a	high	common
hsa-miR-339-5p_R-4	19	TCCCTGTCCTCCAGGAGCT	gp1a	middle	DGF specific
hsa-miR-33a-5p_R-1	20	GTGCATTGTAGTTGCATTGC	gp1a	middle	DGF specific
hsa-miR-363-3p_R-1	21	AATTGCACGGTATCCATCTGT	gp1a	middle	DGF specific
hsa-miR-378a-3p_R-1	21	ACTGGACTTGGAGTCAGAAGG	gp1a	high	DGF specific
hsa-miR-423-3p	23	AGCTCGGTCTGAGGCCCCTCAGT	gp1a	middle	DGF specific
hsa-miR-423-5p	23	TGAGGGGCAGAGAGCGAGACTTT	gp1a	middle	DGF specific
hsa-miR-425-5p_R-1	22	AATGACACGATCACTCCCGTTG	gp1a	middle	DGF specific
hsa-miR-451a_R-1	21	AAACCGTTACCATTACTGAGT	gp1a	high	common
hsa-miR-484_R-1	21	TCAGGCTCAGTCCCCTCCCGA	gp1a	middle	DGF specific
hsa-miR-486-5p	22	TCCTGTACTGAGCTGCCCCGAG	gp1a	high	common
hsa-miR-652-3p_R-1	20	AATGGCGCCACTAGGGTTGT	gp1a	middle	DGF specific
hsa-miR-92a-3p	22	TATTGCACTTGTCCCGGCCTGT	gp1a	high	common
hsa-miR-93-5p	23	CAAAGTGCTGTTCGTGCAGGTAG	gp1a	high	DGF specific
hsa-miR-98-5p	22	TGAGGTAGTAAGTTGTATTGTT	gp1a	middle	DGF specific
hsa-miR-99a-5p_R-1	21	AACCCGTAGATCCGATCTTGT	gp1a	high	DGF specific
hsa-miR-99b-5p	22	CACCCGTAGAACCGACCTTGCG	gp1a	middle	DGF specific
eca-miR-451_L+1R-1_1ss20TA	22	CAAACCGTTACCATTACTGAGT	gp2a	middle	DGF specific
efu-miR-9226_L-4_1ss22GA	18	GTCCCTGTTCGGGCGCCA	gp2a	middle	DGF specific
mmu-miR-3968_L-3_1ss14AT	18	ATCCCACTCCTGACACCA	gp2b	middle	DGF specific
cgr-miR-1260	18	ATCCCACCGCTGCCACCA	gp2b	middle	DGF specific
bta-miR-2478_L-2	18	ATCCCACTTCTGACACCA	gp3	middle	DGF specific
PC-3p-1061_715	18	AGTCCACGATCCCCGTGG	gp4	high	common
PC-3p-2112_237	19	CCCACCACGTTCCCCGTGG	gp4	middle	common
PC-3p-4600_77	19	TTCCACCACGTTCCCGGTG	gp4	middle	ctrl specific
PC-3p-4883_70	18	CACGTTCCCGGGCCGTGG	gp4	middle	common
PC-3p-857_1046	18	ATTCCAACGTTCCCGTGG	gp4	high	common
PC-5p-1596_363	18	GTTCCCGTGGTCCCGTGA	gp4	high	common
PC-5p-3627_109	18	CTCCACCACTTCCCGTGG	gp4	middle	common

gp1a: reads were mapped to miRNAs/pre-miRNAs of specific species in miRBase and the pre-miRNAs were further mapped to genome and EST. gp2a: reads were mapped to miRNAs/pre-miRNAs of selected species in miRBase and the mapped pre-miRNAs were not further mapped to genome, but the reads (and of course the miRNAs of the pre-miRNAs) were mapped to genome. The extended genome sequences from the genome loci may form hairpins. gp2b: reads were mapped to miRNAs/pre-miRNAs of selected species in miRBase and the mapped pre-miRNAs were not further mapped to genome, but the reads (and of course the miRNAs of the pre-miRNAs) were mapped to genome. The extended genome sequences from the genome loci may not form hairpins. gp3: reads were mapped to miRNAs/pre-miRNAs of selected species in miRBase and the mapped pre-miRNAs were not further mapped to genome, and the reads were not mapped to genome either. But the reads were mapped to the miRNAs (Matures). gp4: reads were not mapped to pre-miRNAs of selected species in miRBase. But the reads were mapped to genome and the extended genome sequences from genome may form hairpins.

**Table 4:** The differentially expressed miRNAs between DGF and Ctrl (*P* < 0.05).

miR name	miR sequence	Genome ID	Pre-miRNA sequence	Fold change	log⁡2	*P* value
					(fold change)	
hsa-miR-33a-5p_R-1	GTGCATTGTAGTTGC ATTGC	chr22	ggcagctgtgGTGCATTGTAGTTG CATTGCatgttctggtggtacccatgCA ATGTTTCCACAGTGCATCAca gaggcctg	3.954	1.983	0.007
hsa-miR-98-5p	TGAGGTAGTAAGTT GTATTGTT	chrX	aggattctgctcatgccagggTGAGGTA GTAAGTTGTATTGTTgtggggtag ggatattaggccccaattagaagataactatac aacttactactttccctggtgtgtggcatattca	5.777	2.530	0.024
hsa-miR-151a-5p	TCGAGGAGCTCACA GTCTAGT	chr8	ggggcaaagatgactaaaacacttttcctgccc TCGAGGAGCTCACAGTCTAG TatgtctcatcccctaCTAGACTGAA GCTCCTTGAGGacagggatggtcata ctcacctc	3.375	1.755	0.027

DGF: delayed graft function; Ctrl: control; Fold change: fold change (DGF(mean)/Ctrl(mean));* P* value: DGF versus Ctrl, *t*-test.

## Data Availability

The clinical data of the patients used to support the findings of this study are included within the article. The original sequencing data of the microRNA used to support the findings of this study are available from the corresponding author upon request.
